# Evaluation of Pulse Rate, Oxygen Saturation, and Respiratory Effort after Different Types of Feeding Methods in Preterm Newborns

**DOI:** 10.1155/2022/9962358

**Published:** 2022-06-14

**Authors:** Dipen Vasudev Patel, Dharti Shah, Kunjal A. Kantharia, Mayur K. Shinde, Jaishree Ganjiwale, Kushal Shah, Somashekhar Marutirao Nimbalkar

**Affiliations:** ^1^Department of Neonatology, Pramukhswami Medical College, Shree Krishna Hospital, Charutar Arogya Mandal, Bhaikaka University, Karamsad, Gujarat, India; ^2^Department of Pediatrics, Pramukhswami Medical College, Charutar Arogya Mandal, Bhaikaka University, Shree Krishna Hospital, Karamsad, Gujarat, India; ^3^Central Research Services, Bhaikaka University, Charutar Arogya Mandal, Karamsad, Gujarat, India

## Abstract

**Background:**

During the initial days of hospitalization, preterm newborns are given combinations of breastfeeding, spoon/paladai feeding, and/or gavage feeding. Each method of feeding may have a different effect on vital parameters.

**Objective:**

To study changes in vital parameters in relation to different feeding methods and postmenstrual age (PMA) in preterm newborns. *Study Design*. This prospective observational study was carried out at a tertiary care neonatal unit. *Participants*. Physiologically stable preterm newborns with PMA less than 37 weeks on full enteral feeds were included in the study. *Intervention*. None. *Outcomes*. Respiratory rate (RR), pulse rate (PR), oxygen saturation (SPO2), nasal flaring, and lower chest indrawing were monitored before and up to 3 h after the breastfeeding/spoon (paladai) feeding/gavage feeding or their combinations. These vital parameters were assessed in relation to the feeding methods and PMA groups using ANOVA.

**Results:**

A total of 383 records were analyzed from 110 newborns. No infant developed chest indrawing or nasal flaring after any feeding method. During the 3 h period of monitoring, vital parameters changed significantly except in the gavage feeding group. The mean PR did not change, but the mean RR and SPO2 changed significantly at different PMA.

**Conclusion:**

Vital parameters changed after different types of feeding methods and at different PMA. A further multicentric prospective study is needed to understand the effect of different feeding methods and PMA on vital parameters.

## 1. Introduction

Appropriate feeding is an important component of essential care for every newborn viz. immediate care at birth including resuscitation, breast milk feeding, temperature control, infection prevention, recognition, and response to danger signs [1, 2]. Breast milk is the basic right of the newborn. The methods of feeding are individualized based on gestational age/weight, clinical condition and tolerance of the newborn. Low birth weight newborns who are often premature may not suck effectively and have poor coordination between sucking and swallowing and between swallowing and breathing. Because of this, newborns with gestational age < 32 weeks (<1250 gm) are usually started with gavage feeding followed by spoon/paladai feeding. Newborns between 32 and 34 weeks (1250-1500 gm) usually accept spoon/paladai feeding, and those > 34 weeks (1500-2000 gm) can accept breastfeeding, and some may require spoon/paladai feeding [1, 3].

It is important to ensure safe transition from initial methods of feeding to breastfeeding. The coordination of sucking, swallowing, and breathing is dependent on postconceptional age but may vary from infant to infant depending on the clinical condition [4–7].

Continuous cardiorespiratory monitoring is a vital component of care in preterm neonates. Vital parameters like pulse rate (PR), respiratory rate (RR), and oxygen saturation using pulse oximetry (SPO2) are constantly monitored during the hospital stay. They are under complex physiological control, and while patterns of variation often reflect normal physiology, they may also represent the earliest signs of deterioration [8–12]. Chest indrawing and nasal flaring are also important components of respiratory function assessment [13].

Breast feeding and spoon/paladai feeding are active feeding methods, and gavage feeding is a passive feeding method. During the transition, premature newborns are often given feeds using a combination of these methods. We assume that each method of feeding has different effects on these parameters because of the nature of the feeding method and level of maturity of the cardiorespiratory, central, and peripheral nervous system affecting coordination of sucking, swallowing, and breathing. Some studies have identified changes in heart rate (HR), SPO2, and RR before, during, and after breast feeding, cup-feeding, and bottle feeding in term and late preterm newborns [14–16]. These findings need to be studied further in preterm newborns for the above mentioned different feeding methods when used alone or in combination.

Standard reference ranges of vital parameters in premature neonates are not available. Alonzo et al. provided heart rate norms for preterm neonates between 23 and 34 weeks of gestation and observed irregular changes based on gestational age and post menstrual age (PMA) [17]. Curzi-Dascalova et al. studied changes in respiratory rate based on gestational age and found no significant changes in preterm newborns in respiratory rates [18]. Oxygen saturation studies in newborns are targeted for studying changes after birth and in full term newborns [19, 20]. Shah et al. studied oxygen saturation for late preterm and term newborns but for the first 48 hours only [21]. Thus, there is a need to do further studies to provide mean levels of vital parameters in preterm newborns based on PMA.

The objectives of our study were to evaluate PR, SPO2, RR, and respiratory effort before and after giving feeds using different methods and to evaluate vital parameters based on PMA in stable preterm newborns.

## 2. Methods

### 2.1. Study Type and Study Settings

This was a prospective analytical observational study conducted at Bhaikaka University, India, from April 2020 to September 2021. Sick preterm newborns are admitted to Neonatal Intensive Care Unit (NICU), and stable preterm newborns weighing more than 1500 gm are admitted to Neonatal Intermediate Care Unit. We follow guidelines provided by Sankar et al. for feeding the preterm newborns based on gestational age and tolerance by the newborns [3]. Clinicians, during their routine rounds, decide about the feeding methods for the individual newborns based on clinical examination and feedback from nurses and mothers in accordance with the above guidelines. During the transition from gavage feed to spoon/paladai feed and from spoon/paladai feeding to breastfeeding, two or all these three methods are used in succession for feeding the newborns. So at a time, a newborn may be on “gavage feeding“(GF) or “spoon/paladai feeding” (SF) or “breastfeeding” (BF) or the combination of these feeding methods like “BF followed by SF” or “BF followed by GF” or “SF followed by GF” or “BF followed by SF followed by GF.” Nurses give GF and SF. They also assist mothers to give SF and BF.

### 2.2. Participants

All the physiologically stable preterm newborns with PMA less than 37 weeks who were on full enteral feeds at every 3-hour interval were eligible.

### 2.3. Exclusion Criteria

Infants having feed intolerance, hemodynamic instability, respiratory distress, and bronchopulmonary dysplasia, on invasive/noninvasive ventilatory support or on free flow oxygen were excluded from the study.

### 2.4. Sampling Procedure

The Institutional Ethics Committee of Bhaikaka University, Karamsad, Anand, Gujarat, approved the study. After taking an informed written consent from parents, two study authors evaluated PR, RR, SPO2, nasal flaring, and chest indrawing before and after finishing the feeds. Timing of recording of the parameters was dependent on the availability of either author. The infants included were categorized into the GF, SF, and BF groups or combination of these feeding methods, i.e., BF & SF (BS group), BF & GF (BG group), SF & GF (SG group), and BF & SF & GF (BSG group). The vital parameters were recorded before the feed, immediately after the feed, and at 5 min, 10 min, 15 min, 30 min, 1 hour, 2 hours, and 3 hours after the feeding.

SPO2 and PR were recorded using a pulse oximeter. Pulse oximeter probe was attached to the right upper limb for uniformity. To avoid observer bias, respiratory rate was calculated in the calm newborn over a period of one minute. It is important that newborns should be calm while measuring these parameters as physical efforts/cry makes measurement difficult and also affect them because of increased metabolic demand. Young infants usually breathe faster than older infants and young children. The respiratory rate of a newborn is often more than 50/minute. Therefore, 60 breaths per minute or more is the cutoff used to identify fast breathing in a young infant [22].

During the time of counting respiratory rate, chest indrawing was also looked for. Chest indrawing was considered present if the chest wall went in when he/she breathed in. Nasal flaring was considered present if widening of the nostrils occurred when the newborn breathed in. Routine painful invasive procedure like heel prick was avoided during this 3-hour observation period to avoid stress and effect on viral parameters.

### 2.5. Sample Size

The Central Limit Theorem justifies the use of the normal distribution if the sample size is large enough. If the sample size is greater than 30, it is said to be sufficient empirically. As a result, we considered 30 to be the minimum number of observations required per group for study in the absence of any reference for sample size calculation considering the exploratory nature of the study.

### 2.6. Statistical Analysis

Descriptive analysis was used to define the baseline characteristics of the study participants. Repeated Measure ANOVA was performed using Greenhouse-Geisser Statistics to study change in vital parameters over a period in relation to the feeding method. One-way ANOVA was used to compare baseline vital parameter between PMA groups. *P* values < 0.05 were considered statistically significant. The analysis was performed using STATA (14.2).

## 3. Results

We collected 383 observations from 110 newborns. Of them, there were a total of 43 (39.09%) females. Baseline characteristics of these newborns is provided in Table 1. The median (IQR) age at the time of assessment was 7 (4, 16) days.

**Figure 1:**
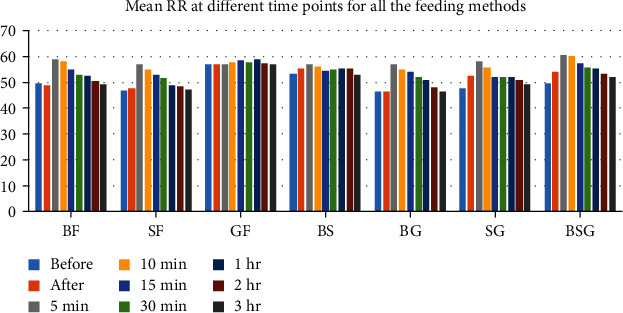
Mean RR at different time points for all the feeding methods.

RR [mean (SD)] at different time points for all the feeding methods is depicted in Table 2. There was rise in RR at 5 min in the majority of groups except the GF group. After 5 min, it reduced gradually towards baseline at 3 hours after feed in the majority of the groups except the SG and BSG groups (Figure 1).

**Figure 2:**
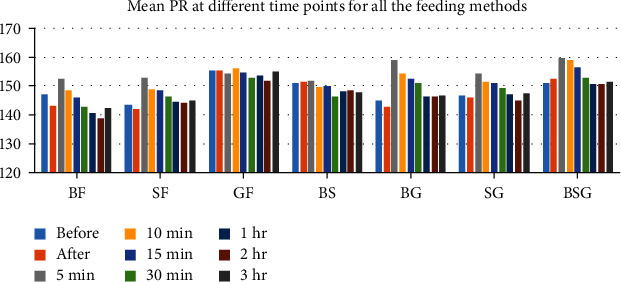
Mean PR at different time points for all the feeding methods.

PR [mean (SD)] at different time points for all the feeding methods is depicted in Table 3. There was rise in PR at 5 min in majority of groups except in the BS and GF groups. After 5 min, it reduced gradually towards baseline at 3 hours after feed in the majority of the groups except in the BF and BS groups where it reduced below the baseline (Figure 2).

**Figure 3:**
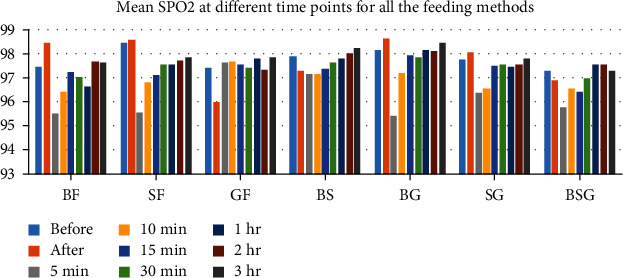
Mean SPO2 at different time points for all the feeding methods.

SPO2 [mean (SD)] at different time points for all the feeding methods is depicted in Table 4. We noticed the trend towards nonsignificant rise in SPO2 immediately after feed in the SF, BG, and SG groups while in the GF, BS, and BSG groups there was nonsignificant reduction. There was a sharp reduction at 5 min in the majority of the groups except GF. Mean SPO2 reached towards baseline at 3 hours after feed in the majority of the groups except the SF group where it was below baseline. In the BF group, the mean SPO2 increased significantly immediately after feed (Figure 3). No significant change in RR, PR, or SPO2 was observed in the GF group.

**Figure 4:**
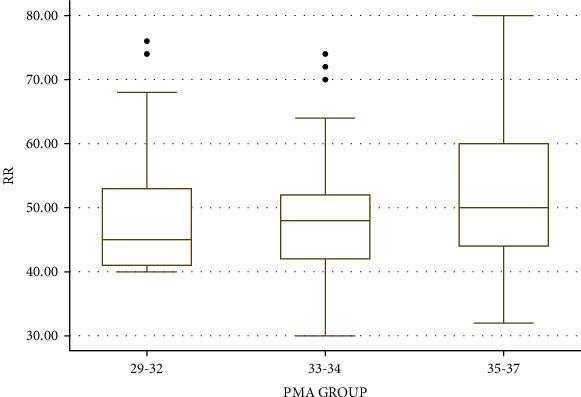
Boxplot showing distribution of RR based on PMA groups.

**Figure 5:**
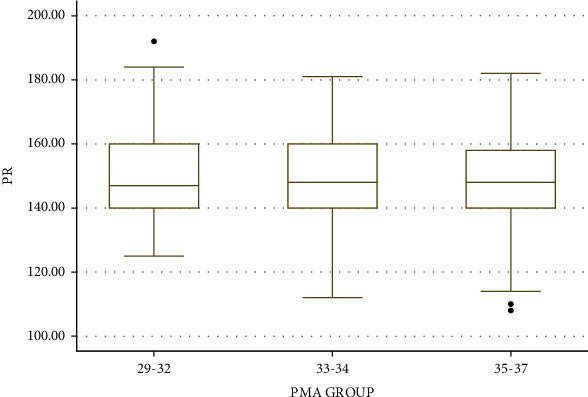
Boxplot showing distribution of PR based on PMA groups.

**Figure 6:**
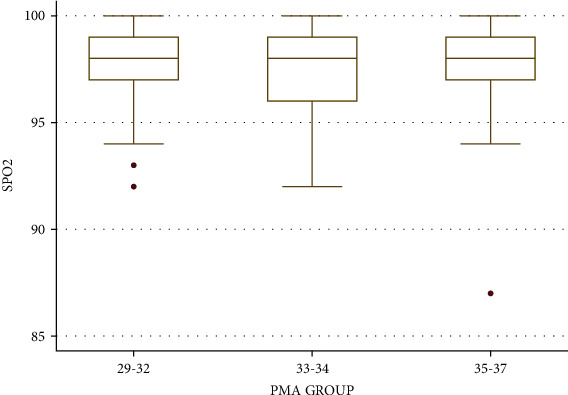
Boxplot showing distribution of SPO2 based on PMA groups.

The Correlation Coefficients between the PR, SPO2, and RR with PMA (from 29 to 37 weeks) were -0.02, 0.03, and 0.18, respectively. Irrespective of the feeding methods and gestational age at birth, mean RR, PR, and SPO2 calculated at baseline period, i.e., before feeding based on different groups of PMA up to 37 weeks, are given in Table 5. One-way ANOVA showed that mean RR and SPO2 varied significantly at different PMA groups. Post hoc analysis showed that RR and SPO2 were significantly higher in the PMA group of 35 to 37 weeks as compared to the PMA group of 33 to 34 weeks. There was no significant change in mean PR between different PMA groups. Distribution of frequencies of RR, PR, and SPO2 is provided in Figures 4–6, respectively. Chest indrawing and nasal flaring did not develop in newborns of any groups after feeding.

## 4. Discussion

Stable preterm newborns on full enteral feeds admitted in neonatal care unit were included in the study. They were categorized into different feeding groups and were evaluated for vital parameters (RR, PR, and SPO2), nasal flaring, and lower chest indrawing before and immediately after finishing the feed and at 5 min, 10 min, 15 min, 30 min, 1 hour, 2 hours, and 3 hours after finishing feeds. Vital parameter changed at different time points after different types of feeding methods in preterm newborns. There was no significant change in all the vital parameters in the GF group at different time points. We did not notice any correlation of SPO2, RR, and PR with PMA, but we observed that mean SPO2, RR, and PR varied significantly at different PMA.

In a study from a tertiary care hospital, Niaz et al. monitored 60 healthy term newborns for HR and SPO2 before, during, and after breastfeeding. SPO2 was lower during breastfeeding than after feeding, and HR was higher during breastfeeding than before feeding. Both were comparable between before and after breastfeeding [14]. In contrast to observations made by the above study, we observed significant rise in SPO2 and drop in PR immediately after feed as compared to before feed in preterm newborns. Thus, PR and SPO2 changed differently in term and preterm neonates. These changes may be because of differences in physical efforts of sucking, maturity of nervous system, cardiovascular system, and lungs between term and preterm newborns [15, 23, 24].

Suiter et al. assessed association between breastfeeding with SPO2 and HR in 22 term newborns at 1 week and 2 months of age. They monitored at every 30 seconds for 5 minutes before oral feeding, during the first 10 minutes of feeding, and the first 10 minutes immediately after feeding. Overall mean SPO2 level was significantly high in 2-month-old infants than 1-week-old infants. SPO2 did not change before, during, and after feeding. The HR increased significantly during breastfeeding as compared to before and after breastfeeding [15]. In our study, we observed that in the breastfeeding group SPO2 significantly increased immediately after feeding and dropped at 5 and 10 min after breastfeeding as compared to before feeding and PR significantly decreased after feeding as compare to before feeding. These findings also suggest that feeding changes the vital parameters differently in term and preterm newborns.

Marinelli et al. enrolled 56 late preterm infants in a prospective, randomized crossover study and compared HR, RR, and SPO2 before and during the feeding using 30 ml medicine cup and bottle feeding. Significant changes occurred in all these parameters between the two feeding methods. HR and RR increased, and SPO2 decreased during both cup and bottle feedings compared to prefeeding baselines. However, they observed more desaturation below 90% and higher heart rates in bottle feeding group at the time of feeding as compared to no changes during cup feeding. Respiratory rate changes were comparable between the groups [16]. We did not monitor vital parameter during feeding, and we used either paladai or spoon in current study. In our study we observed rise in PR at 5 min, 10 min, and 15 min after the spoon feeding as compared to before feeding, and SPO2 was low at all-time points except immediately after the feed, while RR significantly increased at 5 min to 2 hour after feed as compared to before feed. This post feed rise in PR and RR can be explained by activation of the sympathetic system during active swallowing and sucking. Rise in SPO2 immediately after feed may be because of better coordinated respiratory efforts after feeding as compared to immature sucking, swallowing, and breathing coordination during feeding in preterm newborns [25].

In a prospective cohort study from Toronto, Shah et al. monitored SPO2 over 6 h in healthy 20 late-preterm and 40 term neonates during the first 48 h of age and assessed the impact of gestational age. Lower gestation and lower birth weight were associated with higher time spent (25 min versus 13 min) at SPO2 below 90% [21]. We did not monitor percent time spent, but we observed that mean SPO2 was higher in 35 to 37 week PMA group as compared to 33 to 34 week PMA group.

There is limited available information based on PR, RR, and SPO2 reference ranges for the premature neonatal population. Alonzo et al. did a retrospective observational study in premature neonates (23 to 34 weeks) admitted at the University of Virginia to provide reference ranges of HR based on gestation and PMA. There was a slight increase in HR during the initial weeks after birth, followed by a gradual decline with age. They stressed the need of HR reference ranges in the premature neonates for routine monitoring [17]. Similar to this study, in the current study, mean HR in different PMA groups was around 150 beats/minute. We calculated mean vital parameters based on PMA groups irrespective of birth maturity and provided the reference table for user-friendly assessment.

We observed increased SPO2 and RR in newborns at 35-37 weeks PMA. This can be explained by improved central mechanisms like central nervous system development, more mature peripheral reflex pathways, and lung development leading to more regular and unobstructed breathing [24, 26, 27].

The current study compared the vital parameters over a period of 3 hours in relation to different feeding methods. The findings of this study should be considered a pilot study and replicated at different sites for generalization. The findings may have implications in deciding the time of monitoring vital parameters in the preterm newborns. Further studies of comparing variations in vital parameters before feed and at different times after feeds should be done to identify the best time of routine monitoring which has fewer variations in vital parameters.

There were some limitations in this study. All stable preterm newborns were included in the study irrespective of their course during stay in intensive care unit. There were no newborns with PMA below 28 weeks, so we could not generate information for these group of newborns. This was a single center study, so the findings may not be generalized for vital parameters based on PMA groups.

## 5. Conclusion

Vital parameters changed periodically at different time points in different feeding methods except gavage feeding. Mean RR and SPO2 varied significantly at different PMA. Chest indrawing and nasal flaring did not occur after feeds in any group. A further prospective study is needed to strengthen the evidence of effect of feeding methods on vital parameters. Local reference charts should be created for vital parameters based on PMA for routine monitoring in preterm newborns.

## Figures and Tables

**(a):** 

Characteristic	*N*	Mean	SD
Birth weight (grams)	110	1662.80	358
Gestational age (weeks)	110	33.15	2.05
Weight at the time of assessment (grams)	383	1677.02	278
PMA at the time of assessment (weeks)	383	34.42	1.69

**(b):** 

Group	*N*	Mean PMA (weeks)	SD
BF	51	34.93	1.79
SF	63	34.09	1.59
GF	56	33.79	1.93
BS	93	34.88	1.06
BG	31	33.96	2.21
SG	35	34.43	1.17
BSG	36	34.64	2.21

**Table 2:** RR at different time points for all the feeding methods.

	RR	Before	After	5 min	10 min	15 min	30 min	1 h	2 h	3 h
BF	Mean	49.84	49.01	58.98	57.96	55.03	52.90	52.35	50.62	49.25
	SD	9.81	9.16	5.08	5.70	5.546	6.74	6.99	9.46	8.97
	*P* value		0.21	<0.001	<0.001	<0.001	<0.001	0.004	0.269	0.46
SF	Mean	46.76	47.61	57	54.79	52.88	51.58	48.76	48.47	47.14
	SD	9.37	9.92	6.46	6.84	7.55	8.12	8.46	9.33	8.43
	*P* value		0.10	<0.001	<0.001	<0.001	<0.001	0.004	0.034	0.55
GF	Mean	56.80	56.96	56.78	57.75	58.42	57.89	58.78	57.28	56.78
	SD	9.69	7.72	8.44	7.55	8.20	8.33	8.07	7.56	7.95
	*P* value		0.86	0.98	0.431	0.193	0.426	0.129	0.657	0.98
BS	Mean	53.22	55.37	56.83	56.06	54.49	55.01	55.52	55.13	52.73
	SD	9.54	6.94	7.05	8.70	6.91	6.22	7.20	7.27	6.85
	*P* value		0.003	<0.001	<0.001	0.157	0.061	0.016	0.026	0.578
BG	Mean	46.38	46.51	56.83	54.83	53.93	52	50.96	48.03	46.25
	SD	5.89	7.26	4.97	6.14	6.86	6.23	7.62	6.22	5.813
	*P* value		0.90	<0.001	<0.001	<0.001	<0.001	<0.001	0.041	0.852
SG	Mean	47.77	52.45	58.28	55.82	52.11	51.94	52.28	50.74	49.25
	SD	11.11	13.60	10.97	12.58	11.37	10.95	12.29	12.65	10.036
	*P* value		<0.001	<0.001	<0.001	0.002	<0.001	<0.001	<0.001	0.023
BSG	Mean	49.55	54.27	60.50	60	57.38	55.88	55.27	53.33	52
	SD	7.54	9.26	5.56	5.06	5.73	5.79	6.43	6.08	6.43
	*P* value		<0.001	<0.001	<0.001	<0.001	<0.001	<0.001	<0.001	0.028

**Table 3:** PR at different time points for all the feeding methods.

	PR	Before	After	5 min	10 min	15 min	30 min	1 hr	2 hr	3 hr
BF	Mean	146.96	143	152.58	148.33	146.11	142.72	140.47	138.64	142.41
	SD	11.94	9.26	16	13.55	12.66	12.54	13.29	12.98	12.88
	*P* value		0.019	0.098	0.613	0.734	0.088	0.004	0.001	0.006
SF	Mean	143.30	141.95	152.69	148.92	148.66	146.36	144.44	144.25	144.71
	SD	10.23	12.07	11.06	11.37	10.74	11.23	11.09	12.4	12.85
	*P* value		0.176	<0.001	<0.001	<0.001	0.011	0.32	0.504	0.361
GF	Mean	155.33	155.51	154.48	156	154.83	152.82	153.57	151.69	155.08
	SD	13.35	13.61	13.18	14.61	15.89	15.91	14.52	14.87	11.5
	*P* value		0.9	0.618	0.704	0.812	0.203	0.293	0.062	0.882
BS	Mean	150.92	151.48	151.69	149.75	149.89	146.19	148.20	148.67	147.87
	SD	15.1	16.11	16.32	14.7	14.67	15.48	13.56	13.08	12.23
	*P* value		0.659	0.647	0.453	0.518	0.006	0.055	0.152	0.031
BG	Mean	144.70	142.54	159	154.48	152.38	151.19	146.32	146.19	146.58
	SD	15.87	15.28	12.13	11.89	12.3	12.45	11.06	12.32	14.36
	*P*value		0.211	<0.001	<0.001	<0.001	0.007	0.414	0.464	0.161
SG	Mean	146.8	145.85	154.14	151.54	150.88	149.25	146.97	144.74	147.34
	SD	13	14.05	9.97	9.87	11.24	11.16	8.98	10.12	12.2
	*P* value		0.525	0.002	0.004	0.025	0.155	0.899	0.267	0.752
BSG	Mean	150.97	152.66	159.77	159.19	156.38	152.97	150.72	150.5	151.22
	SD	10.9	12.23	9.91	9.46	8.68	7.77	9.91	9.31	9.4
	*P* value		0.313	<0.001	<0.001	0.01	0.264	0.907	0.76	0.876

**Table 4:** SPO2 at different time points for all the feeding methods.

	SPO2	Before	After	5 min	10 min	15 min	30 min	1 h	2 h	3 h
BF	Mean	97.47	98.47	95.52	96.42	97.25	97.04	96.62	97.66	97.62
	SD	1.48	1.08	3.68	2.41	2.11	1.91	2.37	1.54	1.36
	*P* value		<0.001	<0.001	0.009	0.206	0.616	0.084	0.584	0.307
SF	Mean	98.46	98.59	95.56	96.83	97.13	97.56	97.57	97.73	97.86
	SD	1.08	1.01	2.66	2.03	1.93	1.8	1.98	1.73	1.33
	*P* value		0.357	<0.001	<0.001	<0.001	<0.001	0.001	0.002	0.001
GF	Mean	97.43	96	97.64	97.66	97.54	97.41	97.8	97.32	97.84
	SD	2.13	1.96	1.97	2.11	2.38	2.22	1.94	2.5	1.93
	*P* value		0.53	0.438	0.401	0.707	0.946	0.223	0.708	0.1
BS	Mean	97.88	97.3	97.18	97.15	97.37	97.64	97.83	98.01	98.26
	SD	1.99	2.16	2.29	2.64	2.4	2.55	2.33	1.84	1.63
	*P* value		0.093	0.043	0.136	0.245	0.485	1	0.274	0.062
BG	Mean	98.16	98.63	95.41	97.19	97.94	97.84	98.16	98.13	98.47
	SD	1.42	0.838	3.02	1.87	1.85	1.85	1.29	1.41	1.2
	*P*value		0.129	<0.001	0.03	0.629	0.542	0.926	0.724	0.455
SG	Mean	97.77	98.06	96.37	96.57	97.51	97.57	97.46	97.54	97.8
	SD	1.73	1.67	2.34	2.23	2.02	1.85	2	1.65	1.65
	*P* value		0.373	0.009	0.011	0.516	0.56	0.4	0.521	0.922
BSG	Mean	97.28	96.92	95.78	96.53	96.44	96.97	97.53	97.56	97.31
	SD	1.42	1.81	2.23	2.23	1.85	1.69	1.44	1.73	1.21
	*P* value		0.181	0.004	0.119	0.058	0.419	0.469	0.469	0.922

**Table 5:** Vital parameters based on PMA groups.

Parameter	PMA group (weeks)	*N*	Mean (SD)	95% confidence interval for mean		*P* value
RR	29-32	44	48.68 (9.78)	45.7060	51.6576	<0.001
	33-34	122	48.48 (8.32)	46.9909	49.9764	
	35-37	217	52.68 (10.62)	51.2608	54.1033	
PR	29-32	44	150.11 (13.83)	145.9064	154.3209	0.334
	33-34	122	150.25 (13.78)	147.7827	152.7254	
	35-37	217	148.1 (13.83)	146.2552	149.9568	
SPO2	29-32	44	97.86 (1.88)	97.2898	98.4375	0.035
	33-34	122	97.50 (1.69)	97.2049	97.8115	
	35-37	217	98.0 (1.65)	97.7837	98.2256	

## Data Availability

Data is available on reasonable request with the corresponding author.

## Abbreviations

BF: Breastfeeding
GF: Gavage feeding
NICU: Neonatal Intensive Care Unit
PR: Pulse rate
RR: Respiratory rate
SPO2: Pulse oximetry
SF: Spoon/paladai feeding
BS: BF & SF
BG: BF & GF
SG: SF &GF
BSG: BF & SF & GF.
